# Recent Evidence on Bioactive Glass Antimicrobial and Antibiofilm Activity: A Mini-Review

**DOI:** 10.3390/ma11020326

**Published:** 2018-02-24

**Authors:** Lorenzo Drago, Marco Toscano, Marta Bottagisio

**Affiliations:** 1 Laboratory of Clinical Chemistry and Microbiology, IRCCS Galeazzi Orthopaedic Institute, 20161 Milan, Italy; marta.bottagisio@grupposandonato.it; 2 Laboratory of Clinical Microbiology, Department of Biomedical Sciences for Health, University of Milan, 20133 Milan, Italy; toscano.marco1@gmail.com

**Keywords:** silicate glass, surface functionalization, bioactivity, osteostimulation, bone bonding, bone substitute, antibiofilm activity, antibacterial activity

## Abstract

Bone defects caused by trauma or pathological events are major clinical and socioeconomic burdens. Thus, the efforts of regenerative medicine have been focused on the development of non-biodegradable materials resembling bone features. Consequently, the use of bioactive glass as a promising alternative to inert graft materials has been proposed. Bioactive glass is a synthetic silica-based material with excellent mechanical properties able to bond to the host bone tissue. Indeed, when immersed in physiological fluids, bioactive glass reacts, developing an apatite layer on the granule’s surface, playing a key role in the osteogenesis process. Moreover, the contact of bioactive glass with biological fluids results in the increase of osmotic pressure and pH due to the leaching of ions from granules’ surface, thus making the surrounding environment hostile to microbial growth. The bioactive glass antimicrobial activity is effective against a wide selection of aerobic and anaerobic bacteria, either in planktonic or sessile forms. Furthermore, bioglass is able to reduce pathogens’ biofilm production. For the aforementioned reasons, the use of bioactive glass might be a promising solution for the reconstruction of bone defects, as well as for the treatment and eradication of bone infections, characterized by bone necrosis and destruction of the bone structure.

## 1. Overview of Bioactive Glass

Bone defects caused by trauma or related to pathological events are major clinical and socioeconomic burdens doomed to increase along with the global population’s aging trend [1]. During the last 40 years, the efforts of regenerative medicine researchers were focused on the development of a non-biodegradable material resembling bone features, able to support load bearing while restoring physiological tissue function [2].

In 1969, Hench and colleagues proposed the use of bioactive glass as a promising alternative to inert graft materials [2,3]. Bioactive glass is a synthetic silica-based material with excellent mechanical and bone bonding properties, widely used as a bone substitute in grafting procedures [4,5]. Indeed, Hench and colleagues observed that a particular glass composition (Na_2_O–CaO–P_2_O_5_–SiO_2_) was able to form a strong bond with bone that could not be removed without damaging the tied tissue, launching the field of bioactive glass [6]. According to the composition of the glass, which varies depending on the percentage of elements in it, there are many formulations available on the market approved by the United States Food and Drug Administration [2]. Principal bioactive glass formulations are summarized in Table 1.

In particular, it is known that the concentration of SiO_2_ particles influences the bond with the surrounding host tissues; 45–52 wt % SiO_2_ is enough to guarantee bonding to bone and soft tissues, while 52–60 wt % permits bonding to bone only [2,4]. A concentration by weight higher than 60% SiO_2_ enormously slows down the rates of bone bonding, making the glass biologically inert. The first bioactive glass proposed, the 45S5 Bioglass, has a mixture of 25 wt % Na_2_O, 25 wt % CaO, 6 wt % P_2_O_5_, and 45 wt % SiO_2_ [7]. Therefore, the adjective “bioactive” refers to the ability of a material to react in response to physiological stimuli bonding to the host bone tissue [8]. Thanks to the osteostimulation and angiogenic potential, bioactive glass promotes the tissue regeneration needed in the reconstruction of bone defects [9,10]. Hence, different formulations of bioactive glass have been proposed and developed according to their subsequent clinical use. Currently, bioactive glass is successfully used in craniomaxillofacial bone reconstruction, oral, head and neck surgery, spinal procedures, and in the treatment of bone fractures and tumors [11,12,13,14,15,16,17,18].

Numerous applications of bioactive glass other than its use as a graft material have also been investigated, exploiting the material’s features. Indeed, the contact of bioactive glass with biological fluids results in the release of ions from the granules’ surface, leading to an increase in the osmotic pressure and pH and making the surrounding environment hostile for microbial growth without affecting the host tissues [19,20,21,22]. For example, the bioactive glass S53P4 displayed antimicrobial activity against a wide selection of aerobic and anaerobic bacteria in both planktonic and sessile form [23,24]. For the aforementioned reasons, the use of bioactive glass in conjunction with antibiotic therapy has been studied in the management of chronic osteomyelitis with promising results [25,26,27,28,29,30,31]. All the cited studies reported encouraging results and promising clinical outcomes in the use of bioactive glass, mainly thanks to the absence of toxic reactions or adverse effects. Taken together, all these properties makes bioactive glass well suited to the treatment of bone infections, characterized by bone necrosis and destruction of the bone structure.

## 2. Reaction of Bioactive Glass with Body Fluids

**Figure 1:**
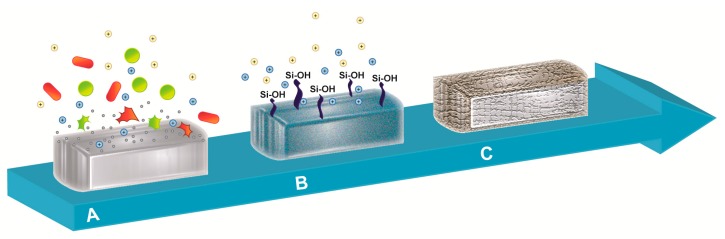
Schematic representation of the reaction upon body fluids contact. The illustration represents (**A**) the leak of ions triggered by the interaction of bioactive glass with body fluid, resulting in bactericidal action against microorganisms, followed by (**B**) the formation of a silica-based layer mediated by the generation of silanol (Si–OH) surface groups and (**C**) the final coating of bioactive glass granule with a mixed carbonated hydroxyapatite layer.

Bioactive glass stimulates various biological responses when immersed in physiological fluids, culminating in the development of a bone-like apatite layer on the granules’ surface, comparable to the mineral phase of bone (Figure 1) [22].

Hench and colleagues described this rapid sequence of chemical reactions as a five-step process [32].

Immediately after the implantation of bioactive glass, (1) the exposure of granules to body fluids causes the exchange of network-modifier ions (e.g., Na^+^, K^+^, Ca^2+^) with H^+^ or H_3_O^+^ ions from surrounding body fluids. This reaction gives rise to an increase in the local pH (from 7 to 10), resulting in an alkaline microenvironment. Additionally, the release of sodium, silica, calcium, and phosphate ions from the bioactive glass surface enhances the salt concentration and the osmotic pressure [33]. These two mechanisms of action efficiently inhibit bacterial growth and, consequently, the adhesion and contamination of implants [24]. The release of protons within the surroundings causes the hydrolysis of the silica groups and, therefore, (2) the formation of silanol (Si–OH) surface groups [34]. Silanol groups also result from the breakage of Si–O–Si bonds due to the increase in the local hydroxyl concentration. These initial phases lead to (3) the development of a silica gel-based layer through the condensation and re-polymerization of the Si–OH groups at the surfaces of granules [35]. Because of the exchange of alkali ions, the silica gel-based layer increases in thickness. Moreover, being negatively charged, (4) the newly formed SiO_2_-based layer acts as a template for the gathering of calcium and phosphate ions already present in the body fluids, creating a stratum rich in amorphous calcium phosphate phase on top of the silica gel [36]. In this phase, (5) the amorphous CaO–P_2_O_5_ phase crystallizes as it bonds to hydroxide and carbonate anions from the surroundings, and gradually transforms into a mixed carbonated hydroxyapatite (HCA) [35]. Within weeks, the HCA layer covers the entire surface of implanted bioactive glass granules, starting the process of osseointegration. Indeed, at the interface, HCA crystals bind to collagen fibrils formed by local osteoblasts, generating a strong bond [2]. Finally, bioactive glass plays a key role in the osteogenesis process due to its osteostimulating properties. Indeed, the inorganic dissolution products of bioactive glass granules stimulate the recruitment of osteoprogenitor cells and their proliferation and differentiation into matrix-producing osteoblasts, which increase the local rate of bone remodeling and the healing of bone [8,36,37].

## 3. Antibacterial Activity of Bioactive Glass

In recent years, there has been increasing interest in the potential antibacterial properties of bioactive glass. Various mechanisms have been proposed regarding their mode of action, such as changes in the environmental pH and osmotic pressure, and “needle-like” sharp glass debris that could potentially damage bacterial cell walls, thus creating hollows and holes on the cell wall and facilitating the penetration of antimicrobial agents in the microbial cytoplasm [38].

The surface reactions described above are not only advantageous for bone regeneration, but also mediate the antibacterial properties of bioactive glass [10,22,23,38,39,40,41].

It has been hypothesized that the antibacterial properties of bioactive glass are due to the increase in local pH following the exchange of sodium ions with protons in body fluids. A shift to a higher alkaline environment is stressful for bacteria, which respond by changing their morphology and ultrastructure, modifying the expression pattern of numerous genes and proteins [42]. Zhang et al. observed that the dissolution of sodium ions leads to an initial increase in the local pH to a value of up to 11 within 8 h, maintaining this high pH value for more than 48 h [22]. Interestingly, the antibacterial activity was observed to decrease when the pH of the media is neutralized, suggesting that this may be a fundamental mechanism mediating the antibacterial action of bioactive glass [38,43]. However, the importance of such a mechanism might be lessened in in vivo conditions, due to buffering of the system. Hence, to mimic a situation closer to the physiological environment, a recent study investigated the antimicrobial activity of bioactive glass S53P4 combined with an autologous bone graft [44], observing its ability to maintain good, though attenuated, in vitro antimicrobial activity in the presence of body fluids and tissues [44]. Although the antibacterial properties of bioactive glass due to the local pH increase have been extensively demonstrated in vitro, little evidence has been reported in an in vivo buffered system [45]. An additional factor contributing to antimicrobial properties is the release of silica, calcium, and phosphate ions, causing perturbations of the membrane potential of bacteria and determining a higher osmotic pressure [8,36,37]. The concentration of solutes within the bacterial cytoplasm is normally higher than that detected in the surrounding environment, resulting in a positive pressure on the cell membrane. A sudden increase in the external solutes concentration causes a rapid water efflux and a pressure drop across the cell membrane, resulting in altered cell size, cell shape, and membrane stress levels [46]. Indeed, a recent study showed that morphological changes (i.e., cell shrinkage, reduction of cell dimensions, and damage to bacterial membranes) are induced in *Staphylococcus epidermidis*, *Acinetobacter baumannii*, and *Klebsiella pneumoniae* strains after incubation with bioactive glass [47].

Bioactive glass has been tested in vitro against a wide variety of aerobic and anaerobic bacteria, showing a fast killing and growth inhibitory effect without selecting for resistance, together with a good activity against biofilm formation, as will be discussed in detail later [26,28,29,30,43,47,48,49,50].

The published studies on the antibacterial properties of bioactive glass are characterized by a wide heterogeneity linked to the bacterial species tested as well as the composition, size, and concentration of bioglass used [21]. Moreover, different methods to assess the antibacterial activity of different types of bioactive glass have been proposed (Table 2).

Bioactive glass shows activity against several clinically important bacterial strains: both Gram-positive and Gram-negative species, and aerobic and anaerobic ones [23,40].

However, the great variability among studies makes it difficult to obtain specific and unambiguous conclusions.

Indeed, to date bioactive glass with different chemical compositions was used across studies (e.g., different percentages of SiO_2_, Na_2_O, CaO, and P_2_O_5_ [40,49]), as well as different particle sizes of bioactive glass, which range from nanometers to a maximum of 0.8 mm. The dimension of particles, in particular, seems to be determinant of the antimicrobial activity. Indeed, the reduction in particle size and the resulting increase in the surface area may enhance the contact of bioglass with the aqueous environment, thus augmenting the diffusion of ions, the local pH, and, lastly, the osmotic pressure [50,51,52]. Finally, the incorporation of bivalent cations such as phosphate (P), copper (Cu), zinc (Zn), strontium (Sr), etc. within the bioactive glass structure has been proposed to improve the antimicrobial effect, while stimulating the bone metabolism, improving the formation of tissue, and inhibiting its resorption by osteoclasts. Moreover, these ions are also known to exert a pivotal role in mineralization and angiogenesis [53].

## 4. Bioactive Glass against Multidrug-Resistant (MDR) Bacteria

The treatment of bone infections is an invasive and expensive procedure, which often requires prolonged hospitalization, numerous surgical procedures, higher risk of complications, and long-term antimicrobial treatment [54,55,56]. Infection leads to various degrees of bone loss, due to the septic process, the related inflammatory reaction, and necessary surgical debridement. The conventional treatment regimen involves surgical debridement of infected tissue and systemic antibiotic administration [57]. Due to the poor accessibility of infected cortical bone to circulating antibiotics, alternative approaches have been developed to enhance drug delivery, including the use of biomaterials to locally administer high doses of therapeutic agents [58,59]. Single antibiotics or combinations of different ones may be loaded into bone cements [60,61,62,63]. Debridement and implantation of antibiotic impregnated polymethylmethacrylate (PMMA) beads is a common procedure, but presents the disadvantage of a second surgical intervention for the removal of the beads [64].

Moreover, the widespread use of antibiotics for several decades has led to an increased prevalence of antibiotic-resistant bacteria, challenging our ability to treat bacterial infections [65,66,67]. The inadequate release kinetics from antibiotic-loaded biomaterials can further favor the development of MDR bacterial strains, making the available commercial antibiotic-loaded bone substitutes ineffective [68].

In this context, the broad-spectrum antimicrobial activity of bioactive glass makes it ideal for the management of bone infections.

Drago et al. first explored the in vitro antibacterial activity of the bioactive glass S53P4 against MDR microorganisms commonly involved in osteomyelitis, showing marked bactericidal activity after 24 h against all species tested [28]. Another recent study assessed the antibacterial activity of gamma-irradiated bioactive glass 45S5 with particle size <45 μm against the MDR microorganisms responsible for bone infections [69]. The results revealed that irradiated 45S5 bioactive glass was effective against the tested strains, with scanning electron microscopy images showing cell shrinkage and membrane damage after exposure of bacteria to bioactive glass [69]. The work by Gholipourmalekabadi et al. investigated the effects of newly synthetized bioactive glass doped with fluoride/silver on the growth of MDR bacteria isolated from patients with burns, finding promising results in 1% silver-containing bioactive glass [70].

The antimicrobial mechanism of action of bioactive glass is completely different from those of traditional antibiotics. Ionic dissolution products from bioactive glass increase the medium pH and osmolarity, creating an inhospitable milieu for bacterial proliferation [71]. As shown in a recent work, bacteria do not seem to be able to adapt to this hostile environment [47]. Such study assessed the in vitro antimicrobial activity of bioactive glass S53P4 against MDR microorganisms involved in bone infections, evaluating its capability to select for resistance in such microorganisms. Repeated exposure did not change minimum inhibitory concentrations (MIC), which were equal to basal values and remained stable after subcultures without antibiotics, demonstrating that the exposure did not lead to a decrease in susceptibility [47]. Moreover, no induction of resistance to bioactive S53P4 was observed during incubation in bactericidal concentrations [47].

## 5. Antibiofilm Activity of Bioactive Glass

Biofilms are microbial communities that can be found on virtually all surfaces, both biotic and abiotic [72]. In recent years, there has been increasing interest in studying biofilms as they are often involved in numerous human infections, from endocarditis to periprosthetic infections [73,74].

Biofilm formation is often associated with the differentiation of multicellular organisms and starts with the attachment of microorganisms to a surface, where they create two layers under the control of specific genes [75]. After an initial phase of cellular division, microbial cells begin producing a polysaccharide matrix that protects the whole biofilm from external agents, ensuring its stability [76].

The last step of biofilm formation is cell detachment, which allows the dissemination of single or clustered cells to other parts of the body [77]. This mechanism leads to an increase in microbial pathogenicity, with a subsequent dissemination of microbial infection and the establishment of new foci of infection [78].

To date, orthopedic infections represent a serious burden due to the high number of hip and knee prostheses that become infected and need surgical replacement [79].

Moreover, the diagnosis of biofilm-associated infections is still difficult and uncertain, as bacteria embedded in the polysaccharide matrix are not detectable by the common analytical methods [74].

Furthermore, microorganisms residing in biofilms are resistant to antimicrobial agents due to the polysaccharide matrix that acts as a physical barrier that slows the penetration and diffusion of antibiotics [74]. Also, the host’s adaptive and innate immune responses are inhibited by the biofilm, which limits the action of polymorphonuclear leukocytes against microbial cells [74].

Consequently, it is of great importance to find new strategies to disrupt biofilm and release microorganisms, improving the diagnostic pathway and increasing the efficacy of antibacterial therapies.

Several methods have been reported in the literature to assess the antibiofilm activity of the bioactive glass to qualitative and quantitative evaluate the biofilm disruption, the biomass thickness, and the viability of bacteria within the matrix (Table 3).

Drago et al. [24] showed the strong anti-biofilm activity of bioactive glass S53P4; indeed, the aforementioned bioactive compound was able to interfere with the biofilm produced by *S. aureus* and *P. aeruginosa* on titanium discs. These two bacterial strains are often involved in periprosthetic infections and are the main biofilm producers with a major clinical impact [24]. Interestingly, the authors highlighted the ability of S53P4 bioglass to reduce the biofilm mass by approximately 80% compared to negative controls, regardless of the bioglass formulation used during the experiment (e.g., powder or granules). However, experiments were performed only in static conditions and, probably, the use of dynamic liquid systems would have reduced the antibiofilm activity observed [22].

Interestingly, the ability of bioglass S53P4 to reduce biofilm produced by *S. aureus* was also shown by Coraça-Huber et al. [52], who highlighted a strong reduction of biofilm mass after it was placed in contact with bioactive glass, as well as a significant decrease in the staphylococci load when planktonic cells were treated with bioglass [52].

Furthermore, the anti-biofilm activity of bioglass S53P4 towards a broad range of microorganisms was underlined in a recent study that underlined the ability of S53P4 to reduce the biofilm produced by other microorganisms of clinical interest, such as *A. baumannii*, *K. pneumoniae*, and *S. epidermidis* [82]. Probably, bioglass S53P4 could reduce the viability of bacterial cells thanks to its antibacterial activity discussed in the previous paragraph.

Finally, the anti-biofilm activity of bioglass seemed to be enhanced when additional antibacterial molecules were incorporated in bioactive glass.

For instance, the combination of bioactive glass with sodium fluoride and triclosan showed a great enhancement of bioglass anti-biofilm activity; indeed, Xu et al. [80] observed an increased susceptibility of bacteria contained in biofilm to antimicrobial agents when treated with fluoride- and/or triclosan-combined bioglass. The treatment, indeed, led to damage to bacterial cells, favoring the access of antimicrobial and anti-biofilm agents to the microbial cytoplasm and blocking glycolysis and the activity of bacterial enzymes [80].

Also, the combination of copper with bioglass was observed to have a strong impact on the biofilm production of *S. epidermidis*, inhibiting bacterial growth and counteracting biofilm formation and dispersion [81].

These promising results may lead to the application of bioactive glass, both alone and in combination with antibacterial compounds as coatings of orthopedic prostheses, making these devices less susceptible to the establishment of bacterial biofilm.

## 6. Conclusions

Bone and joint infections represent an important public health burden, and to date there are few antimicrobial molecules effective against pathogenic microorganisms. Moreover, the increase in MDR bacteria is leading to antibiotic therapy failure and a significant increase in morbidity and mortality.

Bioactive glass shows strong antibacterial effects for a wide selection of aerobic and anaerobic bacteria, due to the increase of pH and osmolarity in the surrounding environment.

No resistance selection has been observed so far, suggesting that bacteria cannot adapt to the hostile milieu created by bioactive glass. In addition, these compounds are able to interfere with biofilm, reducing its mass and improving the diagnostic process and patient outcome, thus representing ideal bone substitutes for the treatment of osteoarticular and prosthetic joint infections.

Numerous studies highlighted the promising features of bioactive glass, often underlining how their efficacy is related to their size or combination with antimicrobial agents able to increase bacteria damage, thus favoring the access of antibacterial and anti-biofilm agents to microbial cytoplasm.

However, little information about specific antibacterial and antibiofilm activity exists, so further studies are needed to deepen this knowledge and improve the use of bioactive glass in eradicating periprosthetic joint infections.

## Figures and Tables

**Table 1:** Composition of bioactive glass. Data are reported as a percentage and refer to the concentration by weight of the compound.

Bioglass	SiO_2_	Na_2_O	CaO	P_2_O_5_	Other
45S5	45	24.5	24.5	6	-
42S5	42.1	26.3	29	2.6	-
S53P4	53	23	20	4	-
55S4	52.1	21.5	23.8	2.6	-
58S	60	0	36	4	-
70S30C	70	30	0	0	-
45S5F	45	24.5	12.25	6	12.5 CaF_2_
40S5B5	40	24.5	24.5	6	5 B_2_O_3_

**Table 2:** The main methods to assess the antibacterial activity of different types of bioactive glass.

Methodology	Methodology Description	References
Bacterial cultivation test	Direct culture of bacteria with powdered bioglass for 1–4 days. The antibacterial activity of bioglass was correlated with the ions concentration and the pH change in the medium.	[22,23,40]
Direct culture	Evaluation of bacterial growth in the presence of bioglass under static and shaking conditions for 24–96 h.	[38,43]
Indirect culture	Evaluation of bacterial growth in medium conditioned with bioglass under static and shaking conditions for 24–96 h.	[38,43]
Indirect culture with adjusted pH	The pH of bioglass-conditioned supernatants was adjusted by adding HCl to give a final pH of 7.2.	[43]
Time-kill curves	The antibacterial activity of bioglass was tested with morselized bone graft to mimic the in vivo buffering-conditions after 0, 24, 48 and 72 h.	[44]

**Table 3:** Antibiofilm activity of bioglass. Table shows the main quantitative and qualitative methods to assess the antibiofilm activity of different types of bioactive glass.

Methodology	Methodology Description	References
Activity against mature biofilm	Following bacterial biofilm formation, titanium discs were placed in direct contact with bioactive glass from 24 h to five days.	[52]
Crystal violet	The biofilm formation on titanium disks was observed by means of a colorimetric assay.	[24]
Confocal Laser Microscopy (CLM)	The biofilm biomass on titanium disks was observed by means of CLM.	[24]
Scanning Electron Microscopy (SEM)	The antibacterial activity against immature biofilm grown on coverslips was assessed by means of SEM.	[80,81]
MTT test	Following biofilm formation on a 96-well plate after incubation with bioglass, the viability of bacteria was evaluated by means of an MTT test.	[81]
